# Mindfulness and CBT: a conceptual integration bridging ancient wisdom and modern cognitive theories of psychopathology

**DOI:** 10.3389/fpsyg.2024.1489798

**Published:** 2024-12-11

**Authors:** Shadi Beshai

**Affiliations:** Department of Psychological Science, Kennesaw State University, Kennesaw, GA, United States

**Keywords:** CBT (cognitive behavioral therapy), mindfulness, mindfulness-based interventions, schema, depression, conceptual integration, dysfunctional attitudes

## Abstract

With the rapid expansion of mindfulness and its incorporation into the “third wave” of Cognitive Behavioral Therapy (CBT), there has been evident confusion about what mindfulness is and how it relates to this broader category of interventions. In this article, I define mindfulness and CBT, and differentiate them while highlighting their substantial overlap. Specifically, I discuss the Buddhist Psychological Model and how it relates to the foundational cognitive model, demonstrating the common threads that run across these seemingly disparate philosophies. I use depression throughout as the exemplar disorder through which these connections are highlighted. This is all in the hope of helping clinicians and scientists see the common ground across these modalities and comprehend how and why mindfulness has come to be associated with the “third wave” of CBT. Ultimately, the aim of this brief article is to showcase the breadth of CBT, its concordance with ancient philosophical thought and wisdom, and to demonstrate why mindfulness has been and continues to be effectively integrated into CBT to address a wide range of mental health concerns and fortify efforts toward wellbeing.

## Introduction

There has been a proliferation of interest in mindfulness in the public and scientific spheres over the last two decades (Van Dam et al., 2018). Mindfulness, or paying attention to present-moment internal and external experiences with openness, acceptance, and curiosity (Chiesa, 2013), is now incorporated in medicine (Goyal et al., 2014), psychology (Keng et al., 2011), education (Meiklejohn et al., 2012), policing (Beshai et al., 2022); military (Jha et al., 2010), and in corporate and commercial settings (Good et al., 2016). This rapid growth of the field of mindfulness has come with discernible challenges. One palpable challenge, especially evident in clinical settings, has been the growing misconceptions around mindfulness, and how it may relate to other established modalities in the field, such as Cognitive Behavioral Therapy. There are several papers that attempt to reconcile cognitive behavioral therapy (CBT) and mindfulness (e.g., Maex, 2011; Purser and Milillo, 2015), as well as papers analyzing the bibliometrics of mindfulness and its cultural footprint (e.g., Karl et al., 2022). Singh et al. (2008) also discuss the broad integration of CBT with mindfulness principles. The authors provide an overview of how mindfulness has been incorporated into mainstream CBT approaches such as Acceptance and Commitment Therapy (ACT), Mindfulness-Based Stress Reduction (MBSR), and Dialectical Behavior Therapy (DBT). There are several published works in which researchers provide an integrated model of mindfulness and operant conditioning principles, extending to CBT (Cayoun, 2011; Cayoun and Shires, 2020; discussed below).

However, to the author’s knowledge, few papers to date have focused primarily on the reconciliation of the theoretical foundations of mindfulness and CBT. Specifically, while existing works have discussed the integration of mindfulness with CBT, there remains a gap in the literature addressing the parallels in the described roles and functions of cognitions as described by both the cognitive model and by the Buddhist Psychological Model (BPM). Accordingly, I attempt to close this gap by conducting a side-by-side analysis of critical aspects of the BPM and demonstrate its alignment with the foundational elements of the cognitive model, as proposed by Beck and Haigh (2014). I relate the origins of suffering as described by the BPM to maladaptive schema formation and activation as described by the cognitive model. Further, I emphasize potential overlaps between the Buddhist architecture of mind and the architecture of the self-referential information processing system proposed by the cognitive model of depression. The intention is to bridge these seemingly different approaches and highlight the overlaps in their foundational hypotheses. This comparison is designed to illustrate how mindfulness, as a third wave approach, can complement and enhance first and second wave approaches in CBT.

In this paper, I also attempt to dispel misconceptions around mindfulness as separate from the umbrella of interventions known as CBT. In doing so, I hope to bring cohesion to the field of CBT. This cohesion aims to provide clinicians and scientists with a clearer understanding of how mindfulness-based approaches can continue to be effectively integrated with traditional CBT techniques, potentially informing more nuanced and effective approaches to research and clinical practice.

## Buddhist roots of mindfulness

It is said that in the 5th century BC, the son of a king in the Indian subcontinent named Siddhartha Gautama wandered out from his carefully curated kingdom, against his ruling father’s wishes, to seek truths regarding the fabric of reality and suffering (Aich, 2013; Maex, 2011). His search was prompted by witnessing several forms of suffering: a sick man, an old man, a dead man, and a holy man who had renounced all worldly pleasures and possessions (an ascetic). Siddhartha was so moved by his observations of these “Four Sights,” that he left his luxurious life behind to seek enlightenment, or a path toward the cessation of suffering. The details of the legend of Siddhartha, who later became known as the Buddha or the Enlightened One, are beyond the scope of this paper. However, it is noteworthy that Siddhartha spent years on this search, which included adoption and mastery of several known religious rituals and practices. These practices included the study of meditation under experienced gurus (teachers) and extreme asceticism and self-denial, which left him weak and malnourished. These extreme practices, which did not lead to the cessation of his suffering, led Siddhartha to the insight that there must be a Middle Way: a balance between extreme indulgence and asceticism. In that moment, as the legend goes, Siddhartha decided to meditate under the Bodhi Tree until he found a way to end suffering. His enlightenment came during this meditation (Laumakis, 2023).

As he sat there meditating, Siddhartha realized that suffering is rooted in the constant grasping (clinging) of the impermanent (Grabovac et al., 2011). Namely, he realized that all people, things, objects, thoughts, emotions, and other states of mind are momentary appearances in consciousness or awareness. Even the permanent, static, unchanging sense of ego we call “self” is simply made up of thoughts, images, emotions, hopes, and other apparitions of mind, which appear only momentarily and ultimately pass away or morph. The Buddha believed that enlightenment lies in the deep understanding and continual “remembrance” of the impermanent and “empty” (Sanskrit: *Śūnyatā*, or the lack of inherent or permanent essence or existence) nature of all things (Van Gordon et al., 2017). For example, the device that you are likely using to read this article is a collection of metal, glass, plastic, rubber, and transistors, which collectively we call “cellphone,” “tablet,” or “laptop.” It is inherently empty of an essential, unchanging component called “cellphone” or “laptop.” The Buddha and, eventually Buddhism held that in order to remember these facts, one must continually practice mindfulness as a mechanism for the remembrance of the impermanence and emptiness of all things.

## The Buddhist psychological model

The Buddhist Psychological Model (BPM; Grabovac et al., 2011) builds a more sophisticated philosophy upon this basic realization of the power of remembrance of the true nature of things. The model suggests that suffering arises when we crave pleasant sensations (e.g., feeling happy) or react with aversion to unpleasant sensations (e.g., feeling sad or anxious), both of which are ultimately fleeting states and experiences. In doing so, we perpetuate suffering. This happens since the mind, due to evolutionary, genetic, cultural, or early life influences, attaches “feeling tones” (Sanskrit: *Vedana*) to all experiences. These feeling tones can be positive, negative, or neutral. While most feeling tones associated with experiences are neutral, hence not likely associated with further mental proliferation or elaboration, positive or negative tones more predictably elicit additional mental proliferation in the form of grasping (“I want this feeling to last”; “I want more of that”) or avoidance (“I hate this feeling”; “Why is this not going away?”; “why am I like this?”). This proliferation begets further cognitive analysis and mental elaboration, which then eventually leads to negative emotional experiences. This locks the experiencer into a vicious cycle of negative/positive tones ➔ avoidance/grasping ➔ mental proliferation ➔ distress ➔ negative/positive tones ➔ avoidance/grasping ➔ mental elaboration ➔ distress, and so on.

Major Depressive Disorder is a disorder typified by the experience of negative moods and/or loss of interest that persists, along with other symptoms (disruptions in sleep and appetite; fatigue; poor concentration; thoughts that one is a failure; thoughts of death or suicide) for 2 weeks or longer (American Psychiatric Association, 2022). The BPM (Grabovac et al., 2011) predicts that all people may experience losses or stressors naturally associated with negative feeling tones; however, in accordance with the model, some may be predisposed, through genetics or early life experiences, to avoid or reject these negative tones, which compounds the negative emotions experienced. For example, in the face of stressors, those prone to depression may think “This stress is intolerable. Why do bad things always happen to me.” When they experience minor losses or upsets, such as a friend canceling lunch plans (which is likely naturalistically or evolutionarily associated with negative feeling tones), they may think “here we go again”; “bad things always happen to me.” They may then ruminate or elaborate on these thoughts in the form of connecting these thoughts with interpretations of self: “friends cancel on me because I am not likable”; “I will never amount to anything.” This then leads to a spiral or activation of further mental proliferative cycles, which can ultimately end in a full-blown depressive episode (Beshai et al., 2011).

In the BPM, mindfulness, which is often defined as “paying attention, in a particular way: on purpose, in the present moment, and non-judgmentally” (Kabat-Zinn, 2003), is believed to be the antidote to this cycle of suffering. Mindfulness is believed to loosen the associations in the chain of causation between feeling tones, mental proliferation, and subsequent emotional suffering. Many models of mindfulness, including Bishop et al.’s (2004) tripartite model and Lindsay and Creswell’s (2017) Monitor and Acceptance Theory, argue that the synergy between the component parts of mindfulness – willful attention regulation to present-moment experience combined with acceptance – creates a cascade of additional ameliorative effects. Chief among these effects are decentering and non-attachment, which are typified by a perspective shift wherein internal and external experiences are viewed from a place of non-attachment (i.e., unclinging) and “objectivity.” This shift is likely what allows for the uncoupling of associative links in the cognitive and emotional cycle of suffering.

The Buddha and others who practice mindfulness meditation observed that if one acceptingly becomes aware of the mind’s reactions to feeling tones and their association with other reactive links in the cycle of suffering, one can break the pattern. Accordingly, suffering requires that one fuse with and “buy into” the thoughts and emotions as important, meaningful, or a reflection of self. From the perspective of the BPM, the power of the mind is in convincing individuals that they are the thinker of the thoughts, and therefore the thoughts are “them” and not the product of conditioning through biology, culture, personal experiences, etc. The more one “buys into” thoughts as meaningful and/or informative of self or current circumstance, the more the links in the chain of suffering tighten and become solidified.

## Cognitive behavioral therapy

Cognitive behavioral therapy (CBT) is an umbrella category of interventions that includes behavioral (e.g., exposure, behavioral activation, systematic desensitization, activity scheduling, contingency management, role-playing, etc.) and cognitive (e.g., cognitive restructuring, Socratic questioning, imagery rescripting, cognitive defusion, cost–benefit analysis, etc.) techniques to address a broad range of psychological disorder symptoms (Beshai et al., 2013; Dobson and Dobson, 2009). The behavioral techniques are known as the “first wave” of CBT, while the cognitive techniques are the “second wave” of CBT. The “third wave” of CBT, which includes mindfulness, is a collection of techniques that broadly emphasize acceptance and compassion approaches (Hofmann and Asmundson, 2008). CBT, especially the cognitive techniques and interventions within, is influenced by Stoic philosophy, which holds that it is not the events *per se* that cause distress, but the interpretations people make of such events (Murguia and Díaz, 2015). The cognitive model asserts that there are constituent parts to people’s experiences, including their behaviors, emotions, physical sensations, and cognitions, which are often reactions to and are activated by their current situation or immediate circumstances (Padesky, 2020). The cognitive mediation hypothesis is central to CBT. This hypothesis asserts that, while changing emotional or physical reactions to situations might not be readily doable, changing other aspects, including behaviors and cognitions, will cause change in the other components of experience (Beshai et al., 2013; Dobson and Dozois, 2021).

Beck’s foundational cognitive model (Beck and Haigh, 2014) builds upon these basic premises of CBT. According to Beck and others (Beshai et al., 2012; Clark et al., 1999), the self-referential information processing system is layered. What I refer to here as the self-referential information processing system differs from the general information processing system in that the former deals in the processing, elaboration, organization, and retrieval of information directly related to self (one’s own thoughts, feelings, behaviors, and experiences). This system involves self-reflection and introspection, and plays a key role in the formation of self-concepts, identity formation, and autobiographical memory.

According to Beck, in the deepest layer of the self-referential information processing system are the schemas, which often arise in reaction to genetic influences or early life experiences. Schemas are often rigid and absolute. They are defined as core beliefs or memory representations about self and the world (Beshai et al., 2012). For example, a patient with depression who experienced neglect in early childhood might come to believe “I am unwanted,” as a byproduct of their early experience. This core belief becomes integrated into the self-concept (collection of core beliefs about self), and is elaborated upon as the child matures. Over time, other beliefs and experiences become assimilated into or associated with this core notion (Wuth et al., 2022). These schemas then give rise to the intermediate level of the self-referential processing system. These often take the form of assumptions or rules for living, otherwise known as dysfunctional attitudes. These are “if-then” statements that stem directly from core beliefs or schemas, and are statements to evaluate one’s experiences. For example, that same patient with the core belief of being unwanted might develop a rule or assumption for living as “if people do not give me most of their time, then this must mean they do not want me in their life.” The evaluation process that happens at the intermediate level is done in the service of the schema; the rules for living are attempting to answer the question: Does this experience align with or challenge the core belief? The attitudes or assumptions at the intermediate level are also rigid, since they stem from already rigid, negatively oriented schemas. Accordingly, they also tend to perpetuate the negatively skewed processing (Chahar Mahali et al., 2020).

In the final layer of the self-referential information processing system, according to Beck and Haigh (2014), are automatic thoughts. These are the running commentary that lie just beneath the threshold of awareness. These are also activated last in the chain of activation stemming from the deeper levels of schemas and attitudes/assumptions. For example, the same patient who was described above might have automatic thoughts related to a romantic interest’s last-minute cancelation of their date such as “They do not like me.”; “They’ll never take me seriously as their significant other.”

According to the cognitive model, this hypothetical patient with early life neglect experiences, which gave rise to “unwanted” core beliefs would then be vulnerable to acute and recurrent depression (Beshai et al., 2011). This is because, as mentioned, schemas are thought to be self-perpetuating as they become more dominant in self-referential processing. More and more situations might activate the “I am unwanted” schema, which leads to further (skewed or biased) activation of rigid assumptions around people’s time and interest, giving rise to negative automatic thoughts, which in turn feed into the core beliefs. Also according to Beck, this type of cognitive vulnerability to depression narrows the interpretation bandwidth in the information processing system. Given the increasingly sophisticated and interconnected nature of the core assumption around being “unwanted.” This leads to more and more situations activating this core belief, leading to narrower and narrower interpretations of experiences, which in turn solidify or reinforce the schema. Indeed, there is substantial evidence that people with heightened symptoms of depression exhibit several biases and interpretive errors that tend to reinforce dysfunctional core beliefs and further assert their dominance in the self-referential information processing cycle (Beshai et al., 2014; Gotlib and Joormann, 2010).

By the time patients develop depression, their negative thoughts have become all too familiar, and hence the accuracy of these thoughts goes unquestioned (Begg et al., 1992). Cognitive restructuring works to alleviate symptoms by drawing attention to the skewed nature of automatic thoughts and dysfunctional attitudes, hence (a) slowing down the cycle from schema activation to automatic thoughts, (b) blocking the assimilation of new experiences into the existing schemas, as well as potentially, (c) reorganizing and disintegrating previously assimilated experiences from the schema, hence reducing their dominance in the self-referential information processing system (Dozois and Dobson, 2001).

## Bridging the gaps

At this point, I hope the reader can begin to draw strong linkages between the philosophical and theoretical roots of mindfulness and those of CBT. Feeling tones, which are the initial link in the causal chain of suffering according to the BPM, set off the activation of additional mental proliferation through craving or aversion. In the CBT model, feeling tones, or initial emotional reactions to events or situations, become habitually associated with schemas and schematic activation along the layered self-referential processing system. The pairing of feeling tones with schemas depends on the ultimate meaning this schema holds for “self” and “self-concept,” and the sprawling and interconnected nature of the schema or core belief.

In CBT, the process of meaning generation, where patients interpret events based on their schemas, parallels the craving or avoidance patterns described in the BPM. In CBT, when an event activates a schema, it generates meaning that aligns with the schema’s content. This meaning often leads to emotional responses and behaviors that reinforce the schema. Similarly, in BPM, when a feeling tone arises, it can trigger craving (for positive tones) or avoidance (for negative tones), leading to mental proliferation. In both models, these processes (meaning generation in CBT and craving/avoidance in BPM) are deeply connected to core beliefs or schemas, serving to maintain and reinforce them over time.

For instance, if a hypothetical patient who is vulnerable to depression values deep personal connections and views them as core to self-concept, this patient will come to interpret even minor setbacks toward the goal of connection (e.g., last-minute cancelation of a date or lunch) as a threat to self-concept. Accordingly, the disappointment from a canceled plan becomes linked to a negative core belief embedded within a larger system of core self-values, triggering a negative feeling tone. This negative tone is then avoided due to its perceived implications for self-worth. The interpretation cycle becomes more rigid with continual activation of the core schema around being “unlovable” or “unwanted,” since this schema becomes a dominant node which is further entrenched in a larger self-schema within the self-referential information processing system. Once this occurs, any negative or positive feeling tones associated with this schema will be associated with a reactive need to avoid or cling to such feeling tones, given their perceived threat or relevance to self (Beshai et al., 2011; Teasdale, 1988).

The connectivity between layers of the information processing system predicted by Beck and Haigh (2014), Beshai et al. (2012, 2016), and Clark and Beck (1999) is akin to the chain of proliferation described by the BPM: Feeling tones met with clinging or aversion then give rise to further elaborative thought (schema activation), which then activates additional layers (attitudes; automatic thoughts) and so on. In both models, this reactive cycle of activation and elaboration leads to suffering or psychological distress.

In both the BPM and CBT, thoughts are believed to be central in the cycle of suffering. In the BPM, thoughts are believed to arise in reaction to clinging or aversion to initial feeling tones, which then engender further reactive cognitions, emotional states, and physiological reactions. In CBT, thoughts are believed to link context or situations with further activation of layers in the information processing system. BPM emphasizes that continual observation of these reactive thought patterns with the qualities of mindfulness–acceptance, openness, curiosity–will ultimately break the cycle of reactivity. In CBT, the power of thoughts or meaning imbued in them is systematically questioned through cognitive restructuring or Socratic questioning.

Ultimately, however, both of these modalities emphasize that thoughts, for the most part, think themselves through genetic, cultural, or early life conditioning. Also, that thoughts may not have any ultimate meaning, nor is a reaction to them warranted or healthy. Accordingly, this is why mindfulness fits squarely into the larger umbrella of CBT, as it deemphasizes meaning placed on thoughts by teaching clients that (a) thoughts are part of an elaborative reactivity cycle that occurs ultimately through no volitional control (they are conditioned); (b) reactivity to thoughts gives power to them and perpetuates and solidifies the cycle, and (c) escape from this cycle is possible through accepting and open awareness. This narrative is also consistent with evidence suggesting that across CBT and other psychotherapies, the meta-mechanism associated with change is related to loosening rigid beliefs around self-narratives in which patients get stuck (Salkovskis et al., 2023).

## Mindfulness and CBT: where they differ

In addition to the obvious differences in their approach – CBT with its emphasis on challenging and dismantling negative and biased cognitions through rational inquiry, and the emphasis of mindfulness on disempowering cognitions through breaking the reactivity cycle that fuels them– there are deeper philosophical differences between mindfulness and CBT. Mindfulness has been stripped of its Buddhist roots and pruned to fit into Western psychological frameworks, such as CBT (Purser and Milillo, 2015). This was a deliberate choice by early adopters of mindfulness, such as Kabat-Zinn (2003) to broaden its appeal among secular Western patients and practitioners. However, and despite these efforts, mindfulness remains deeply rooted in Buddhist and Eastern contemplative philosophies. Given this Buddhist connection, mindfulness possesses elements which make it unique compared to the philosophy that underpins CBT and other modalities forged in a primarily Western context. For example, from a Buddhist perspective, and in addition to reducing suffering, the ultimate goals of the practice of mindfulness are deeply spiritual. Through the cultivation of mindfulness, the Buddha believed that liberation lies in the erosion of the belief in a static, unchanging view of “self” as the “CEO” of experiences (or self as “in control” of internal and external realities and experiences). As mentioned above, Buddhist and other contemplative thinkers see “self” as another fleeting experience that is ultimately empty of an “essence” and one that is based on the conditions of mental elaboration. The static “CEO” view of self, as is consistent with Western philosophies including CBT, is believed to perpetuate suffering from the perspective of the BPM, as people tend to cling to certain standards or conditions for self-worth (Worthwhile self = “happy” self; Worthwhile self as “wealthy” or “attractive” or “satisfied” self). Clinging to these self-standards likely leads to suffering, as these are unrealistic and rigid standards that often do not take into account contexts nor can be achieved indefinitely or permanently. This suspicion of a static self-concept is not present in CBT nor is discussed or elaborated upon in first-generation mindfulness-based interventions (Van Gordon and Shonin, 2020). Second-generation mindfulness interventions have now begun to reintegrate Buddhist notions, such as no-self or Anatta and “emptiness” back into Western applications of mindfulness (Van Gordon and Shonin, 2020).

CBT is a Western intervention very much concerned with symptom diminution as its ultimate goal. There is no emphasis in CBT on deep philosophical or spiritual transformation (Beshai et al., 2013). CBT also views self as agentic and separate or independent from others, which is also in contrast to views of self held in Buddhist interpretations of mindfulness. That is, in Buddhism, self, much like anything else, is interdependent on the existence of other conditions, including ancestors, other people in their social network, and the natural world (Grabovac et al., 2011).

Further, and given its Buddhist and contemplative roots, mindfulness is imbued with ethics which transcend the aims of classical forms of therapy, such as CBT. For example, some researchers have argued that “Right Mindfulness” connotes not just the accepting quality of attention to present-moment experiences, but an emphasis on a deeper connection to prosocial behaviors and compassionate relationships with self, others (including all living things), and the environment (Baer, 2015; Feldman and Kuyken, 2019). Interestingly, Phang and Oei (2012) proposed a Meta-Mindfulness approach. Within this approach, the authors advocated for the integration of Buddhist principles into CBT. They argue for expanding CBT to include Buddhist ethical and emotional dimensions, such as Right Speech (ethical communication), Right Effort (balanced energy and perseverance), and Right Concentration (focused mental training). These are critical components of the Noble Eightfold Path in Buddhist teachings.

Further, mindfulness-based interventions actively encourage and expect patients to develop their skills through cultivating a personal mindfulness practice (Parsons et al., 2017). For example, trainees of first-generation mindfulness-based interventions, such as Mindfulness-Based Stress Reduction and Mindfulness-Based Cognitive Therapy, are encouraged to engage in mindfulness practice for 45 min a day, and are expected to maintain their practice after the conclusion of the intervention (Crane et al., 2017). Similarly, as part of their certification and accreditation, mindfulness trainers and practitioners spend considerable time in meditative practice. For example, MBSR facilitators are required to complete hundreds of hours of mindfulness meditation prior to achieving their certification (Crane et al., 2012). These facilitators, much like the patients, are expected to maintain their own mindfulness practice and to embody mindfulness-related skills and attitudes. This is in stark contrast to the training model in CBT, which does not require practitioners to engage in personal practice of CBT techniques or skills. This distinction highlights a fundamental difference in the training models of CBT and mindfulness, as the latter is focused on learning and embodiment of related attitudes, while the former emphasizes conceptual and pedagogical approaches (Dobson and Dobson, 2009).

Finally, in the CBT model, there are discernible layers to the information processing system (e.g., schemas, attitudes, automatic thoughts) that interact and are activated in a hierarchical manner. The BPM describes several aspects of consciousness (Grabovac et al., 2011); however, these are not demarcated as distinct layers and can be characterized as a fluid, continuous stream of moment-to-moment awareness. In CBT, interventions and techniques are aimed at specific layers, whereas in the BPM, present-moment awareness is enough to slow the “stream” and disrupt its flow.

## Integration of mindfulness in cognitive behavioral protocols

Given the parallels between the theoretical foundations of mindfulness, as expressed by the BPM, and core elements of CBT, many protocols have integrated these perspectives to enhance their therapeutic effects. This integration allows for a synergy that strengthens the impact of both approaches. In this section, I will discuss two pioneering protocols: Mindfulness-Based Cognitive Therapy (MBCT; Segal et al., 2002) and Mindfulness-Integrated CBT (MiCBT; Cayoun, 2011). While I only discuss two examples of this integration below, as they are especially pertinent to depression, there are several other examples of this effective integration of mindfulness and CBT. Examples include Acceptance and Commitment Therapy (ACT), Mindfulness-Based Stress Reduction, Dialectical Behavior Therapy, among others.

### Mindfulness-based cognitive therapy (MBCT)

Mindfulness-based cognitive therapy is a first-generation mindfulness-based intervention, derived from its predecessor, Mindfulness-Based Stress Reduction (Crane et al., 2017). Given that depression often follows a lifelong course (Beshai et al., 2011; Bockting et al., 2015), MBCT was specifically developed to address relapse and recurrence of depression. Although originally designed for recurrent depression, MBCT has since been adapted to treat a wide range of psychological disorders, including acute major depression, generalized anxiety, social anxiety, and eating disorders. It is noteworthy that MBCT has demonstrated its effectiveness in reducing relapse and recurrence in depression (Kuyken et al., 2016), and shows great promise for the remediation of several other disorders. Much like its predecessor, MBCT is delivered in groups over an eight-week program, with two-hour sessions.

The design of MBCT was theoretically driven, specifically targeting differential activation in depression (Teasdale, 1988). Differential activation refers to the strengthening of associations between mood and depressogenic cognitions. Simply put, with each successive episode of depression, even normative dips in mood tend to elicit negative automatic thoughts, dysfunctional attitudes, and ruminative thinking. In MBCT, mindfulness is integrated with cognitive-behavioral principles to help patients with a history of recurrent depression recognize this link between mood and cognition. Over time, patients are theorized to develop decentering, where they view mood changes and negative thoughts from a neutral, meta-perspective. Cultivation of decentering is further theorized to weaken the associations of mood and cognition, and hence reduce the automaticity of this pattern. Thus, patients use mindful awareness to recognize their thoughts and observe their transient nature. Given MBCT’s original focus on reducing relapse and recurrence, patients learn to understand the signs and patterns of relapse and develop skills to help them maintain well-being over longer periods.

While classic forms of CBT might emphasize cognitive restructuring and direct challenges to depressogenic cognitions, MBCT encourages accepting, non-reactive awareness of such cognitions. Further, MBCT emphasizes self-awareness, insight, and disruption of processes related to dysfunctional cognitions. On the other hand, classic CBT is more problem-focused, and emphasizes the content of cognitions.

### Mindfulness-integrated cognitive behavioral therapy (MiCBT)

Mindfulness-integrated cognitive therapy (MiCBT) is a second-generation mindfulness-based intervention. Developed by Cayoun (2011) and Cayoun et al. (2018), MiCBT is a transdiagnostic intervention that integrates mindfulness with classic CBT techniques, such as exposure and cognitive restructuring. Importantly, MiCBT retains the Buddhist influences and contemplative roots of mindfulness throughout the integration process.

In their Co-emergence Model of Reinforcement (CMR), Cayoun and Shires (2020) discussed the importance of interoception – awareness and perception of internal states – as a driver for emotional disorders. The researchers argued that impairments in interoceptive awareness, and the pairing of interoceptive cues with maladaptive thinking and behavioral patterns coupled with reactivity and avoidance are key mechanisms in the perpetuation of depression and anxiety. Accordingly, cultivation of mindfulness, and in turn, improved interoceptive awareness and equanimity – balance and non-reactivity to interoceptive cues – can function as transdiagnostic mechanisms to remediate a wide range of psychological conditions.

The process of MiCBT evolves through several stages. It begins with intrapersonal regulation (Personal Stage), where mindfulness skills are honed to cultivate interoceptive, metacognitive, and bodily awareness. This progresses into behavioral regulation, or the Exposure Stage, where trainees learn to use mindfulness to sit with uncomfortable emotions and thoughts. The third stage, interpersonal regulation, applies mindful awareness to social interactions, improving assertiveness, managing relationship conflict, and enhancing interpersonal understanding. Finally, in the transpersonal regulation, or Empathic Stage, trainees cultivate compassion and ethics, fostering interconnectedness and empathy toward both self and others (Cayoun, 2011).

Key mechanisms of MiCBT include metacognitive and interoceptive awareness, and equanimity, defined as adopting a neutral, non-reactive stance toward both pleasant and unpleasant experiences (Francis et al., 2024). For example, equanimity is believed to play a role in regulating emotions through the balanced receptivity of interoceptive cues. These described mechanisms, cultivated through mindfulness, are theorized to synergize with classical CBT techniques like exposure and cognitive restructuring. This integration enhances the holistic nature of CBT, as individuals learn to address both the content of cognitions and the process of relating to daily experiences.

Therein lies a key difference between classic CBT and MiCBT. CBT emphasizes active vigilance toward and change of depressogenic cognitions; accordingly, patients are encouraged to actively confront negative automatic cognitions and dysfunctional attitudes. By contrast, one of the goals of MiCBT is to cultivate equanimity, or a neutral stance to external and internal experiences, including negative cognition (Cayoun, 2011; Frances et al., 2020). Equanimity is believed to work synergistically with interoceptive awareness to disrupt the conditioned reactivity to body sensations and other interoceptive cues, hence slowing and ultimately disrupting the cycle of distress. There is promising evidence suggesting MiCBT works to reduce depression and anxiety, and does so through the proposed mechanism of increasing equanimity (Frances et al., 2020; Francis et al., 2022).

## Clinical implications

Consistent with the goals of this paper, I am hoping CBT practitioners can now fully understand the relevance of mindfulness and third wave techniques for their CBT practice. The theoretical integration of CBT and mindfulness facilitates a sense of cohesion and continuity among CBT practitioners who may see mindfulness as a distinct practice from CBT. Ultimately, as demonstrated, the BPM and cognitive model are well-aligned with one another. This alignment should inspire excitement about the flexibility of CBT and the breadth of its technical repertoire.

Given the alignment of mindfulness with the broader CBT model, I recommend that clinical trainees be systematically trained in mindfulness theories and applications alongside their typical training in the quintessential CBT model. In this training, the development of collaborative and dynamic case conceptualizations should be cultivated (Kuyken et al., 2016). The selection of appropriate CBT techniques should follow a sound case conceptualization process. Further, and despite an implied dichotomy of choosing second versus third wave techniques for particular clients, some clients might benefit from the integration of these techniques. Indeed, psychological flexibility–the ability to respond to situations in a way that is consistent with personal values–is a central construct in third wave interventions such as ACT (Doorley et al., 2020). Psychological flexibility is broad, and includes the strategic deployment of coping and regulation resources to meet dynamic environmental contexts (Kashdan and Rottenberg, 2010). Accordingly, aiding patients in the flexible deployment of first, second, and third wave techniques might results in substantial and sustainable gains.

Some aspects of mindfulness might even enhance or be synergistic with aspects of traditional, second wave CBT. For instance, there is substantial evidence that mindfulness is associated with better cognitive reappraisal (Garland et al., 2011, 2013). Further, mindfulness seems to be associated with improved executive functioning capacities, including enhanced executive attention (Lin et al., 2018), working memory (Zhou et al., 2020), and cognitive control (Aguerre et al., 2020). All of these aspects are critical in the successful completion of cognitive restructuring exercises. Accordingly, mindfulness-based techniques could be viewed as a potential complement to classic cognitive techniques in the CBT repertoire.

Similarly, aspects of mindfulness can also be synergistic with first wave techniques in CBT. For example, enhanced body awareness is believed to be a consequence and mechanism of mindfulness training in reducing symptoms of distress (Hölzel et al., 2011). Body awareness may function to improve tolerability and effectiveness of first wave techniques, such as exposure. Indeed, there is substantial evidence that mindfulness training may enhance processes (e.g., extinction learning) associated with exposure therapy (Curreri et al., 2020; Treanor, 2011). Further, according to Shapiro et al. (2006), other key mechanisms of mindfulness include value clarification, self-regulation, cognitive and behavioral regulation, and exposure (or openness to experiences). All of these mechanisms are highly consistent with behavior monitoring techniques, and can facilitate behavior regulation consistent with endemic meaning, as predicted by the Mindfulness-to-Meaning theory (Garland et al., 2015).

## Conclusion

In this paper, I argued that mindfulness, or paying attention to present-moment experiences with attitudes of openness, acceptance, and curiosity, has several philosophical and theoretical overlaps with CBT. This was done to provide context as to why mindfulness is incorporated in the “third wave” of CBT. Third wave techniques, much like second, also emphasize the power of self-referential processing or cognitions. Third wave techniques provide an alternative approach to addressing negative cognitions. They complement second wave classic cognitive techniques (e.g., restructuring) in aiding patients to break the reactive, cyclical nature of negative thoughts. This paper was written in the hope of demystifying mindfulness and demonstrating to clinicians the cohesiveness of the larger CBT model and the breadth of its interventions.
